# A three source capture–recapture study of fatal injuries in Iran

**DOI:** 10.5249/jivr.vo112i2.1170

**Published:** 2020-07

**Authors:** Zahra Ghodsi, Soheil Saadat, Abdolrazagh Barzegar, Vali Baigi, Vafa Rahimi-Movaghar, Mohammadreza Zafarghandi, Ardeshir Sheikhazadi, Payman Salamati

**Affiliations:** ^*a*^ Sina Trauma and Surgery Research Center, Tehran University of Medical Sciences, Tehran, Iran.; ^*b*^ Department of Midwifery, Tuyserkan Branch, Islamic Azad University, Tuyserkan, Iran.; ^*c*^ Legal Medicine Research Center, Legal Medicine Organization, Tehran, Iran.; ^*d*^ Forensic Medicine Department, Faculty of Medicine, Tehran University of Medical Sciences, Tehran, Iran.

**Keywords:** Completeness, Injuries, Mortality, Registration, Iran

## Abstract

**Background:**

Well-functioning health systems and effective preventive measures require registering the exact number and valid data of fatal injuries. The present study aimed to determine the completeness of fatal injuries reported by LMO with the use of the capture-recapture method and finding the reasons for those unregistered fatal injuries in Hamedan County.

**Methods:**

This cross-sectional study was conducted in Hamadan County from 22 August 2015 to 21 August 2016. The completeness of fatal injuries reported by LMO, as the main source of fatal injuries was estimated with the employ of the capture-recapture method including Health Department and Police. Log-linear modeling was used for statistical analysis. The number of fatal injuries that probably had not been detected in any three sources was estimated by using the GENLOG command.

**Results:**

A total of 451 fatal injuries were registered in LMO for one year. The registries were included different amounts of detailed information from at least five variables in the Emergency Medical System (EMS) up to all detailed information in the LMO and Health Department. More fatal injuries occurred in males than females at all ages and the two-sex difference spectrum was wider between about 20 to 45 years old. Among cases of LMO, we found 29 unreported deaths. Therefore, the completeness of reported fatal injuries by LMO was estimated to be 86.9%.

**Conclusions:**

Fatal injuries are under-reported by the main source of this type of death in Iran. Identification of fundamental causes, integrated death registry system, and using a standard cause of death classification are needed to promote the registration of fatal injuries.

## Introduction

As a preventable public health concern in recent years,^1^ fatal injuries account for 8.43% of the total global deaths.^2^ Fatal injuries are responsible for approximately 4.7 million cut short lives worldwide annually^3^ and occur at a high rate in low- and middle-income countries.^4^ Injuries constitute 14.8% of all causes of death in Iran;^5^ and among them, road traffic crashes (RTC) with a significant proportion of injury-related deaths were ranked as the third leading cause of death in 2015 compared to eighth rank globally.^2^ External causes of death are related to the substantial economic burden of lost productivity due to premature mortalities and are a large drain on the health care system.^6-8^

The evaluation of statistical values arising from multiple data sources regularly is necessary to ensure that the objectives of the program - providing critical information to health policymakers and planners, resource allocation, and priority setting- are being met, and in this regard, even research priorities should be partly based on the goal of preventing events and improve health. The completeness of mortality statistics undoubtedly has a direct impact in responding to these needs and health system performance.^9,10^ High-quality death registration systems, through providing reliable, accurate data and all available measures provide a starting point for policymakers to design effective strategies and meet the needs of health sector reform programs.^9^ Only 87 of 194 countries have a high-quality or medium-quality death data report system.^11^ In low and middle-income countries, poor data registry and high level of under-reporting account as two important factors in fatal injury registration systems.^12,13^ Iran is among the median quality report countries of cause-of-death statistics and there is no accurate and comprehensive fatal registry system across the country.^11^ A comparison of fatal injuries registration sources creates a comprehensive snapshot of fatal injuries at the national level.^9^ Capture-recapture is a straightforward statistical method that evaluates completeness when there is no single best data source examines some of the data gaps and delivers an accurate estimate of incidence and prevalence of fatal injuries in a short time.^14,15^ There are multiple injury-related deaths data sources in Iran for generating fatal mortality statistics.^16^ Among them, the Legal Medicine Organization (LMO) is the main source for the detection of external causes of death^17^ and the gold standard source for fatal injuries recording in the country. So, the main concern here is that if LMO captures all fatal injuries? If the fatal injuries are under-reported, how many deaths from fatal injuries did LMO report? The question remains are the deaths from fatal injuries assigned to another cause? To answer the questions, the present study aimed to determine the completeness of fatal injuries reported by LMO with the use of the capture-recapture method and finding the reasons for those unregistered fatal injuries in Hamedan County.

## Methods 

This study was conducted in Hamadan County which is consisted of Hamadan, Maryanaj, Jouraqan, and Qahavand cities and the related rural areas, located in the west of Iran, with a population of 651,821 (0.86% of Iran's population).^18^ The study was conducted on data collected from 22 August 2015 to 21 August 2016. Data were reviewed in July 2017 and included all registered fatal injuries. 

The death registration system in Iran is multisource and each source records one or more types of all deaths. LMO is the main source for registration of fatal injuries nationwide. The capture source was established and expanded across the country from 1993. By law, all fatal injuries must be referred to as the LMO to detect the exact cause of death. LMO in Iran is responsible for issuing fatal injuries death certificates. Therefore, LMO is the most reliable source for these records and the gold standard source in this way.^17^ LMO as the main source registers a wide range of variables including full name, age, sex, date of injury, date of death, place of injury, place of death, cause of death, and other characteristics based on death certificate.^19^

Department of Health: Ministry of Health has established a "Death Registration System" (DRS) to the whole of the country. Death causes are classified according to ICD 10 guidelines in this system. The system registers all overall deaths include those fatalities that occurred on the scene, after admission into the medical facilities as well as those who had been reported by “rural health houses”. The later records all fatalities by a population-based approach in the rural area. The Health Departments are obliged to collect in-hospital deaths and report to the DRS regularly.^17^ EMS provides free pre-hospital medical care by means of 2200 bases throughout the country. After providing essential medical care to stabilize the patients, EMS transfers the patients into the relevant medical center if present at injury or death place.^20^ It reports all registered fatal injuries to the Health Department, too. Police data source: Police refers to the scene of all major crashes. Police officers record any traffic causality and mortality. The police report includes a place of the crash, involved vehicle, road and driver characteristics.^21^ Reports of Police are obtained from two sources; traffic crashes occurring intra city is recorded in traffic police statistics, while interurban crashes on roads between the city and the surrounding villages are recorded as road police statistics. 

After the Health Department and EMS data combining, a three capture-recapture approach was utilized to estimate the extent of under-reporting in the LMO dataset in Hamadan County. To examine the completeness of the LMO dataset, we studied if there have been any traumatic death in the Traffic police and Department of Health dataset that was not recorded in the LMO dataset. All the resources considered hospital deaths as recommended by the World Health Organization up to one month after the accident.^22^ We extracted the external causes of deaths for all the registered and unregistered deceased based on death certificates recorded.

There were more than two recorded sources in this study, so, log-linear modeling was used to estimate the number of deaths not recorded by any of the sources. In this model, the estimates were created considering the correlation and heterogeneity of different sources. The HILOGLINEAR command in SPSS 21.0 was used to find out the best model for log-linear analysis, based on the goodness of fit if the different models. Then by using the GENLOG command, we estimated the number of fatal injuries that probably had not been detected in any of the above-mentioned data sources.

## Results

During the study period, there were 451 registered fatal injuries by LMO. There were 29 fatal injuries reported by the Health Department and Police that were not captured by LMO. Table 1 shows the sex; age; causes of death; and the differences among LMO registered and unregistered fatal injuries. In two groups, most deaths occurred at the age of about 20 to 45 years. Also, most unregistered of the deceased were seventy years old or more. The results also showed most of the unregistered deaths occurred among women.

The difference in the distribution of causes of death was observed in two groups of fatal injuries recorded by LMO (451 deceased) and unreported (29 deceased) groups, in which the falls as a cause of death was a large percentage of unreported group (37 %), it was 7.1% in registered group. In the registered group, 44.1% of fatal injuries resulted from death due to MVC, while MVC deaths were 10.3% in the unreported group. This suggests more register deaths from MVC (Table 1).

**Table 1:** The age and sex distribution and cause of fatal injuries in registered cases in LMO (N=451) and unregistered (N= 29).

	Present (451)	Absent (29)	Total
Age, mean (SD), year	38.72 (20.2)	55.79 (25.2)	39.75 (20.9)
**Gender N(%)**			
Men	363 (80.5)	20 (69.0)	389 (79.8)
Women	88 (19.5)	9 (31.0)	97 (20.2)
**Cause of Death N(%)**			
Motor Vehicle Crashes	199 (44.1)	3 (10.3)	202 (42.1)
Falls from height	32 (7.1)	9 (31.0)	41 (8.5)
Falls (same level)	0 (0.0)	2 (6.9)	2 (0.4)
Cold weapon	8 (1.8)	0 (0.0)	8 (1.7)
Drowning	3 (0.7)	2 (6.9)	5 (1.0)
Burn	20 (4.4)	0 (0.0)	20 (4.2)
Drug poisoning	28 (6.2)	0 (0.0)	28 (5.8)
Poison	32 (7.1)	0 (0.0)	32 (6.7)
Suffocation	2 (0.4)	0 (0.0)	2 (0.4)
Hanging	42 (9.3)	1 (3.4)	43 (9.0)
Electrocution	5 (1.1)	0 (0.0)	5 (1.0)
Firearm	8 (1.8)	0 (0.0)	8 (1.7)
Explosion	2 (0.4)	0 (0.0)	2 (0.4)
Hypoxia	3 (0.7)	1 (3.4)	4 (0.8)
Drug abuse	59 (13.1)	5 (17.2)	64 (13.3)
Hard Hit	0 (0.0)	1 (3.4)	1 (0.2)
Drug side effect	0 (0.0)	3 (10.3)	3 (0.6)
Other	6 (1.3)	2 (6.9)	8 (1.7)
Missing	2 (0.4)	0 (0.0)	2 (0.4)

The study results show whereas all death certificates of registered deceased LMO were issued by coroners, whereas among under-reporting deaths eight certificate deaths were issued by other doctors, verbal autopsy questionnaires were issued for eleven deceased by a health volunteer (in Iran known as Behverz) at rural health houses, and three deaths were unknown in terms of any death certificate, death certificates were issued for the other rest of twenty-nine under-reported deaths. 

**Figure 1:**
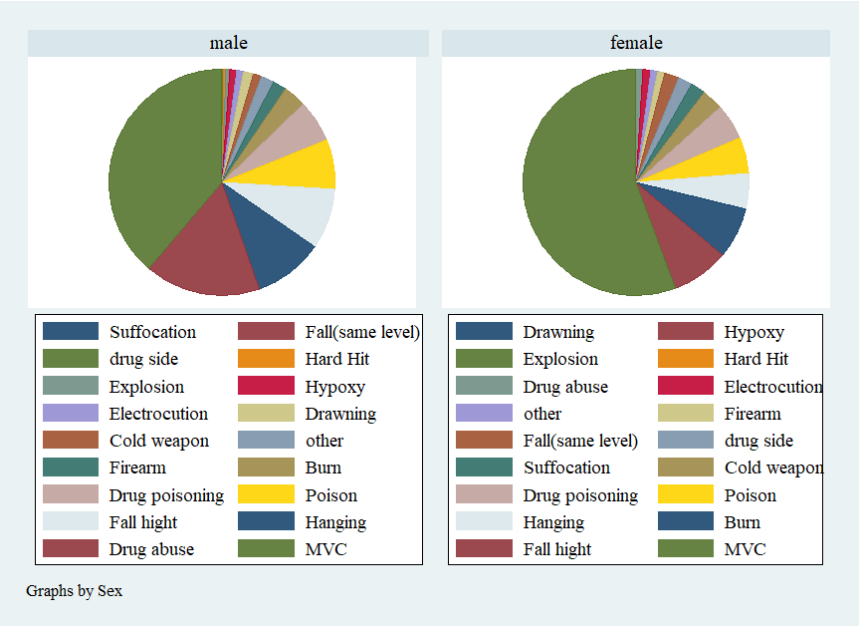
The cause of fatal injuries in registered cases in LMO (N=451) and unregistered (N= 29) by sexMCV: Motor Vehicle Crashes, drug side: drug side effect

Figure 1 shows the six common causes of death differences in both sexes. As the figure shows, the most common cause of death based on LMO classification,^23^ was motor vehicle crashes with a higher incidence in women. The second cause of death was drug abuse in men whereas among women falls accounted as the second cause of death. 

The overall number of fatal injuries cases in Hamadan County reported by LMO was 451 cases. Both the Health Department and EMS reported 238 fatal injuries. The Traffic police had registered 81 traffic fatalities. There were 29 unreported by LMO. Table 2 represents the distribution of detected injury fatalities in different sources. 

**Table 2:** Cases overlapping in the Legal Medicine Organization, Health Department, and Police.

				LMO	
				Present	Absent
Health Department	Present	Police	Present	54	0
			Absent	156	28
	Absent	Police	Present	26	1
			Absent	215	0

Based on the log-linear analysis, it was estimated that 39 (95% confidence interval: 33-79) fatalities would be hidden from all the three sources of data. Therefore, the coverage of the LMO is estimated as follows:

$$ \frac{451}{(451+29+39)}=86.9\%(84.05-89.75) $$

## Discussion

The present study assessed the completeness of LMO, the main source for fatal injuries and by means of the capture-recapture method in Hamedan County. Also, it focused on the fact that if the external causes of fatal injuries are under-reported by LMO, are deaths from fatal injuries assigned to another cause or LMO missing deaths. 

With the use of the capture-recapture method, the study discovered an underestimating about 13 percent of true fatal injuries cases in the LMO. The sources were selected based on for assumptions of the method. To meet the first assumption of the capture-recapture of a closed population, the present study was limited to a one-year period when all samples were selected at the same time and led to limit appropriate estimations of death in Hamadan County fatal injuries registration sources. To provide the second assumption of independence, log-linear methods were used to estimate account for organization dependencies in which the fatal injuries data sources should not subset from the others. In the present study, all samples had the same probability of being captured that it met the third assumption of the capture-recapture which is the homogeneity of reporting probability. Accuracy and availability of sufficient information in each data source in order to match capability is the fourth assumption.^15,24^ In order to avoid matching error, an accurate comparison was conducted for all three sources with common variables. Department of Health was approximately the same coverage of LMO variables included details of information so that after merging the two sources of deceased information, a high degree of matching was captured. Mortality under-reporting is a common finding especially in developing countries.^12,15,25^ Khorasani-Zavareh et al. in the estimation of the completeness of the registration of Fatal Road Traffic Injuries (FRTIs) in eastern Azerbaijan, using the capture-recapture method, ascertain 65% estimated deaths in LMO and death registration system (DRS). The difference between other road users could be a reason; DRS uses verbal autopsy for road users other than pedestrians whereas the coroners issue them in the Legal Medicine System. In addition, two systems have different classifications of road users.^15^ In another study by Abegaz et al., using the same method in Ethiopia on the completeness of registration of FRTIs, the results showed neither Police nor hospital sources provided accurate coverage of FRTIs so that two above sources reported only 57.4-60% and 31.5-33.4% of deaths, respectively.^12^ In Jazayeri et al. study to estimate the prevalence of spinal cord injury (SCI) in Iran, the sensitivity of the Welfare Organization led to the underestimation of the samples. However, it was in an acceptable range at the national level.^13^ Marzban et al., in their study showed that the cancer mortality rate using the capture-recapture method was significantly higher than the reported by routine recording in Iran.^26^

We found more death from fatal injuries than what LMO reported so that there were twenty-nine cases reported by the Health Department and Police that were not registered by LMO. The most cause of death in unregistered deaths was due to falls from height and the same level. Also, the cause of death for three deceased in the unregistered group was due to drug side effects. Neither falls from the same level nor were drug side effects not classified as a cause of deaths based on LMO classification. In addition except for two deceased, all of the fatal injuries due to falls were aged more than 70 years. The use of an integrated cause of death classification and an accurately assigning cause of death could help to the issue.

In the present study, the number of unreported fatal injuries to LMO was reported by Behvarzes in rural areas or other physicians. Whether rural deaths occur in a hospital or elsewhere, Bhvarz applies a verbal autopsy questionnaire for the deceased that lived in the village. Failure to review regular forms by a physician and earlier registration of the forms in the health system despite the death certificate issued at the hospital can be considered as one of the reasons for a mistake in determining the underlying cause of death. Failure to correctly identify the cause of death by the physician or the lack of familiarity with the principle standards for completing the death certificate, low quality of cause of death coding, reporting, and recording of deaths, could be considered as the main reasons for under-reporting.^17^ These issues could probably be solved with stimulating the hospital staff responded to the timely enter death certificate and check the recorded form type, more accurate surveillance by hospital manager, applying automatic methods to determine the cause of death, help to accurately record deaths, and determine the exact cause of death. According to the World Health Organization report, developed countries generally have a high-quality death record system.^27^

In Mathers et al. study, Canada, Japan, Iceland, and Cuba were among countries with a high-quality death registration system with completeness of 100 percent. In these countries, ideally, the death record system captures all deaths by calculating the age-specific death rate and cause of death.^28^ According to World Health Statistics, in Iran completeness of death registration with detailed cause-of-death information is about 90 percent in 2015.11 Iran uses the ICD-10 classification for causes of deaths and is compared with other countries of Eastern Mediterranean Regions with the use of GBD causes of diseases and injury classification.^29^

Despite the emphasis on training physicians in completing the death certificate in Iran, failure to complete and record the accurate cause of death certificates of death is still one of the main causes of the error in the death registration system.^17^ Under-reported fatal injuries could create this idea in health policymakers to produce more accurate estimation and use of an integrated cause of death classification to enrich epidemiological purposes. An integrated fatal injury registration system is recommended to perform more accurate correction and precise registers and adequately support policy development in the coming years.

A limitation of the study was that it is related to a single region, Hamadan County, therefor fatal injuries could not be generalized at the national level.

## Conclusion

The present study showed about 13 percent of fatal injuries underestimating by LMO with the use of a capture-recapture method. LMO is the main source of fatal injuries reports and a gold standard source for this type of death. It has an almost good function because most of the unreported fatal injuries in the study were due to wrongly assigning causes of deaths from fatal injuries to other cases. Under-reporting of injury-related mortality could create an idea in health policymakers to produce a more accurate and integrated classification of deaths in sources that capture deaths. Also, an integrated fatal injury registration system is recommended to perform more accurate correction and precise registers and adequately support policy development in the coming years as Hatamabadi et al study.^30^ Repetition of the study at the subnational level and the other regions of the country are recommended. It seems to consider investment in causes of under-reporting by health sectors to help safety interventions and preventable programs.

**Acknowledgment**

This manuscript is based on the thesis of Zahra Ghodsi under the supervision of Professor Payman Salamati to achieve a Ph.D. degree. The researcher appreciates the personnel of LMO, the Health Department, EMS, and the Police who have collaborated in this study.
